# Trends and Disruptions in Antiretroviral Treatment Enrollment in Haiti, 2018–2024

**DOI:** 10.4269/ajtmh.25-0491

**Published:** 2026-03-31

**Authors:** Chris Delcher, Cheolwoo Park, Jungjun Bae, Daniella Myriam Pierre, Jackyvens Camille, Fred J. Noel, Ermane G. Robin

**Affiliations:** ^1^ Institute for Pharmaceutical Outcomes and Policy, Department of Pharmacy Practice and Science, College of Pharmacy, University of Kentucky, Lexington, Kentucky, USA;; ^2^ Department of Pharmaceutical Outcomes and Policy, College of Pharmacy, University of Florida, Gainesville, Florida, USA;; ^3^ Department of Mathematical Sciences, Korea Advanced Institute of Science and Technology, Daejeon, Republic of South Korea;; ^4^ Department of Epidemiology and Biostatistics, Graduate School of Public Health and Health Policy, City University of New York, New York, USA;; ^5^ Ayiti Analytics, Port-au-Prince, Haiti;; ^6^ Programme National de Lutte Contre les IST/VIH/SIDA, Unité de Coordination des Maladies Infectieuses et Transmissibles, Ministère de la Santé Publique et de la Population, Port-au-Prince, Haiti

## Abstract

Haiti’s unprecedented sociopolitical upheavals have severely impacted health care services. Limited research exists on HIV clinic operations under such strain. We examined the potential interruptions to antiretroviral treatment (ART) provision through discrete time periods using Haiti’s national HIV surveillance system. We analyzed weekly count data from HIV clinics (January 2018–December 2024). Time segments were bounded by the COVID-19 emergency period (April 2020–September 2020), hospital strikes (November 2020), the presidential assassination (July 2021), protests over increases in fuel prices (September 2022), and intensifying gang violence (March 2024). We accounted for autocorrelation in weekly time series and measured immediate, level, and trend changes in new ART enrollment. We triangulated this series with Google’s weekly cell phone mobility reports to cross-validate our event selection. In Haiti, ART enrollment declined 25% from 13,304 (CY2018) to 11,773 (CY2024) people per year (mean = 222 people/week in 2024). Only the fuel protests had an immediate impact on ART enrollment (β = −111.1 [95% CI: −179.3, −42.9]) and this was isolated to the largest clinical electronic medical record. Mean weekly ART enrollment in most periods was lower. Trend visualization suggests that historical patterns of disruption followed by numerical rebound may have changed after the gang violence in 2024. ART enrollment declined substantially in Haiti from 2018 to 2024. Future work should discern whether the epidemiology of HIV transmission is changing after accounting for these health systems disruptions.

## INTRODUCTION

Health care systems worldwide experienced severe disruptions to HIV services during the emergency period of the COVID-19 pandemic, raising concerns about treatment continuity for people living with HIV (PLHIV).^1^^,^^2^ In low- and middle-income countries (LMICs) like Haiti, maintaining antiretroviral treatment (ART) continuity for PLHIV was further complicated by preexisting structural vulnerabilities and resource constraints.

Haiti already faced substantial health care delivery challenges but recurring sociopolitical unrest, gang violence, and insecurity exacerbated barriers to care in the post–COVID-19 era.^3^ In July 2025, the United Nations warned of the possibility of “total collapse of state authority.”^4^ These ongoing conditions clearly pose additional risks to HIV care continuity beyond those caused by the pandemic itself.^5^

Haiti’s HIV epidemic is characterized by a moderate but persistent burden. According to UNAIDS, the adult HIV prevalence (age 15–49 years) is estimated at around 1.6%, with a higher burden among women (around 2.1%). Recent estimates suggest an annual incidence of approximately 0.7% among adults age 15–49 years, and more than 150,000 PLHIV in Haiti. According to the most recent programmatic cascade, approximately 87% of PLHIV are on ART, and of those on treatment, around 74% have achieved viral suppression. Although HIV services are available across all 10 departments in Haiti, persistent challenges remain in equitable access, timely diagnosis, and treatment retention, especially in urban centers affected by violence and unrest.^6^ According to the Haiti Ministry of Health, 86% of PLHIV with a viral load test are virally suppressed.^7^

Previous studies have documented varied impacts of the COVID-19 pandemic on HIV services across different global regions, highlighting reductions in ART initiation and adherence as well as disruptions in HIV testing and viral load monitoring.^8^^–^^13^ For instance, South Africa and Botswana experienced considerable declines in ART initiations during lockdown periods, especially in urban and larger health care facilities.^8^^–^^10^ Similarly, the Philippines saw substantial decreases in ART initiations during early pandemic months,^14^ and countries such as Ethiopia and Malawi reported disruptions in ART initiation and adherence coupled with increased HIV-related mortality.^11^^,^^12^^,^^14^ Studies from West Africa indicated disruptions in viral load monitoring, although ART initiation rates showed resilience in some contexts.^13^ These disruptions underscore the vulnerability of HIV care delivery systems during health and social emergencies, particularly in resource-limited settings.^15^^,^^16^ A meta-analysis found that HIV testing rates declined by 37%.^17^ Patients in Haiti have directly reported interruptions to health care because of civil demonstrations and forced migration out of Haiti’s capital, Port-au-Prince (PAP).^18^

In Haiti, early adaptive strategies during the pandemic included increases in multi-month ART dispensing and community-based ART distribution. ART prescriptions for ≥6 months increased after the onset of the pandemic though patient medication pickups appeared to decline.^5^ Minimizing wait times, allowing more flexibility for home versus clinic visits, and other modifications to the standard of HIV care have been a by-product of extreme instability.^19^

Beyond the pandemic, Haiti experienced significant additional disruptions due to political instability, including the assassination of the Haitian president in July 2021 and widespread civil unrest, which have disrupted public infrastructure and health services. These events have been linked to health facility closures, particularly in PAP, where more than half of PLHIV are located. Previous studies have also showed substantial reductions in healthcare utilization during periods of political instability, emphasizing the vulnerability of HIV services to both health crises, socio-political disruptions, and natural disasters.^20^ These studies have also documented resiliency in HIV services such as the rebound experienced in Haiti after the devastating 2010 earthquake.^21^

Our study aims to assess both the immediate and longer-term effects of the emergency period of the COVID-19 pandemic and subsequent episodes of major social disruptions on ART enrollment trends in Haiti.

## MATERIALS AND METHODS

### Data sources.

We used data from the national HIV case-based surveillance system, the Haitian Active Longitudinal Tracking of HIV database (Suivi Actif Longitudinal du VIH/SIDA en Haïti [SALVH]), operated by the Ministry of Public Health and Population (MSPP) since 2008 and comprehensively described elsewhere.^7^^,^^22^^–^^24^ In brief, there are three electronic medical records (EMR) systems used in Haiti to support HIV patient monitoring, clinical data collection, and reporting activities to SALVH.^25^ These include 1) the iSanté EMR, a collaboration between MSPP, Centre Haïtien pour le Renforcement du Système de Santé (CHARESS), and I-Tech at the University of Washington in the state of Washington, which supports HIV care at MSPP-affiliated clinics nationwide^26^^,^^27^, 2) the Haitian Group for the Study of Kaposi’s Sarcoma and Opportunistic Infections (GHESKIO) EMR, developed specifically to support comprehensive HIV care coordination, patient monitoring, and research at interconnected GHESKIO centers in PAP^28^, and 3) Partners in Health (PIH) – Zanmi Lasante EMR, a web-based platform designed to manage HIV patient data, clinical outcomes, and medical drug supply across 12 rural clinics in Haiti’s Central Plateau region.^29^ In our study, we examined the two central facilities operated by GHESKIO located in PAP, Institut National de Laboratoire et Recherche (INLR) and Institut des Maladies Infectieuses et Sante de la Reproduction (IMIS).^30^

Daily ART enrollment counts, aggregated to the weekly level, from SALVH covered the period January 2018–December 2024. We used the first date of ART enrollment for patients who were classified as either active or lost to follow-up.

This study was reviewed and received ethics approval from the National Bioethics Committee in Haiti (reference: 1718–37) and the Institutional Review Board at the University of Kentucky (reference: IRB201702830). We received a full waiver of informed consent, and the study was approved as expedited because the data were collected originally for nonresearch purposes.

### Count of new patients on ART.

To identify and deduplicate patient records from those newly enrolled in ART during the study period, we selected the facility with the earliest HIV case report date. If missing, we selected the earliest of the ART enrollment or first ART prescription pickup dates. Haiti adopted a “test and start” strategy in July 2016 whereby medical staff attempt to start ART treatment on the same day as HIV diagnosis. The strategy has continued to be successful even during the severe instability.^19^^,^^20^

### Major disruptions selection.

We primarily compiled a list of dates representing impactful or disruptive governmental shutdowns from in-country MSPP staff and US embassy in Haiti website alerts (https://ht.usembassy.gov/category/alert/). Our team decided that the analytical emphasis was on the effects associated with the COVID-19 emergency period from March 2020 (Week 11) to September 2020, the hospital strikes of November 2020 (Week 47–48), the presidential assassination of July 2021 (Week 27–28), the fuel price strikes in PAP beginning in September 2022 (Week 37–44), and the intensifying gang violence in late February 2024 (Week 9–10).^31^ Because the hospital strikes occurred so close to the end of the COVID-19 emergency period, we used November 2020 as the end point of that segment for modeling. The emergency period was defined as the pandemic declaration by the World Health Organization on March 11, 2020.^2^

### Google COVID-19 community mobility.

To triangulate trends and events with external measures of population mobility, we examined cell phone mobility data from Haiti obtained from Google’s COVID-19 mobility reports at https://www.google.com/covid19/mobility/. Google only made this data publicly available from March 2020 to October 2022. We visualized the percentage change in time spent in the workplace relative to the baseline period just before the COVID-19 emergency as an indicator of potential social disruption or large-scale work stoppages. Country data were available at the daily level, which we averaged for weekly analysis. We used this data descriptively and did not statistically integrate this series into our models.

## STATISTICAL ANALYSES

We selected the best fitting autoregressive integrated moving average (ARIMA) models for weekly counts of ART enrollment for 1) Haiti overall, 2) aggregated into two regions (e.g., PAP, Outside of PAP), and 3) each of the three EMR systems (GHESKIO, iSanté, and PIH). These autoregressive models were used to account for temporal correlation (if necessary) in the weekly time series and test for immediate changes in our outcome and level trends during the segmented time periods above. We did not attempt to comprehensively tune all models, because our goal was to reasonably account for the variability prior to statistical testing and strengthen the association with the hypothesized disruptive events and improve estimate precision. The R package auto.arima and SAS 9.4 (SAS Institute, Inc.) software were used for all analyses.^32^

## RESULTS

For calendar year 2018, the start of the study period, there were 13,304 newly enrolled patients on ART in Haiti (mean = 251 people/week). Enrollment increased to 15,743 in 2019 (+18.3%) but eventually declined by 25% to 11,773 in 2024 (mean = 222 people/week). This decline in weekly enrollment was sharpest for GHESKIO over the entire study period at 62.9%. (21/week in 2018 to 8/week in 2024). ISanté, GHESKIO, and PIH comprised 82.1%, 6.1%, and 11.8%, respectively, of the national ART enrollment between 2018 and 2024 (*n* = 94,112). Table 1 shows ART enrollment counts and percentage change by year and week for Haiti and each clinical EMR system.

**Table 1:** Annual and weekly counts with year-over-year percentage change of ART enrollment for Haiti and the primary clinical electronic medical records, 2018–2024 from iSanté, GHESKIO, and PIH – Zanmi Lasante

Suivi Actif Longitudinal du VIH/SIDA en Haïti (SALVH)																
	Electronic Medical Record															
	HAITI				GHESKIO				iSanté				PIH			
	Annual		Weekly		Annual		Weekly		Annual		Weekly		Annual		Weekly	
Calendar Year	Total	%	Mean	%	Total	%	Mean	%	Total	%	Mean	%	Total	%	Mean	%
2018	13,304	N/A	251	N/A	1,127	N/A	21	N/A	10,566	N/A	201	N/A	1,611	N/A	30	N/A
2019	15,743	18%	297	18%	1,087	−4%	21	−4%	12,436	18%	233	16%	2,220	38%	42	38%
2020	14,721	−6%	278	−6%	824	−24%	16	−24%	12,204	−2%	230	−1%	1,693	−24%	32	−24%
2021	12,551	−15%	237	−15%	692	−16%	13	−16%	10,573	−13%	201	−13%	1,286	−24%	24	−24%
2022	13,012	4%	250	6%	765	11%	15	13%	10,884	3%	208	4%	1,363	6%	26	8%
2023	13,008	0%	250	0%	772	1%	15	1%	10,898	0%	206	−1%	1,338	−2%	26	−2%
2024	11,773	−9%	222	−11%	435	−44%	8	−45%	9,700	−11%	183	−11%	1,638	22%	31	20%
All	94,112	–	255	–	5,702	–	15	–	77,261	–	209	–	11,149	–	30	–

ART = antiretroviral treatment; GHESKIO = Haitian Group for the Study of Kaposi’s Sarcoma and Opportunistic Infections; PIH = Partners in Health.

### Haiti, PAP, and other areas.

**Figure 1.**
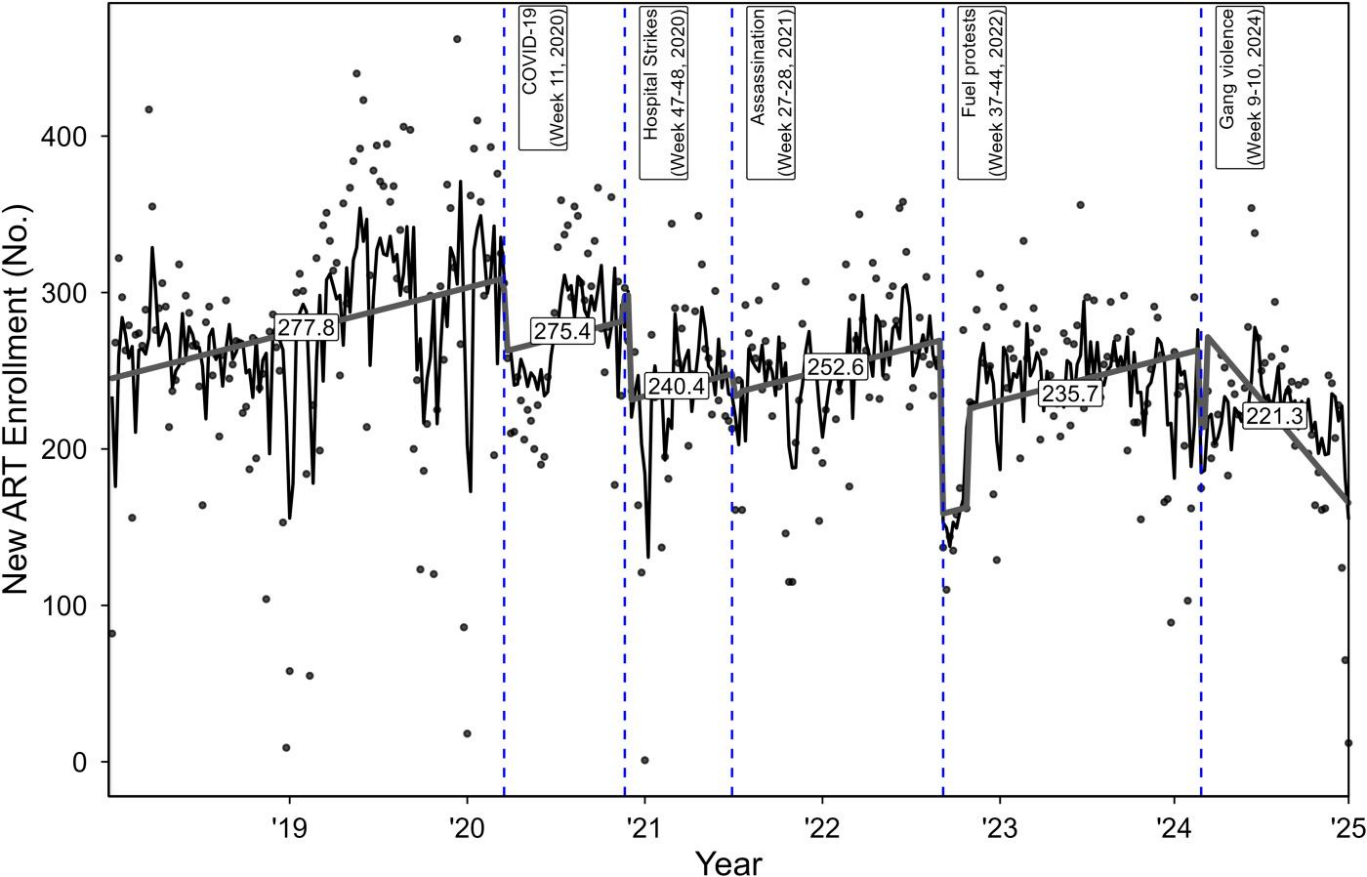
Weekly number of people enrolled in antiretroviral therapy (ART) in Haiti. The black nonlinear lines represent fitted autoregressive integrated moving average models (ARIMA), and points are actual weekly counts. Lines within time periods are slope trends visualized from the ARIMA models with mean weekly counts labeled.

Table 2 shows regression coefficients from the ARIMA models for the immediate disruptions, level, and trend changes for each time period for Haiti and EMRs organized by location around the PAP area. ARIMA specifications are shown in the table notes. Figure 1 shows weekly enrollment counts, linear trends (for visual effect) and means (labeled) throughout segments of the study period for these areas. For Haiti, only the fuel protests had an immediate and statistically significant impact on ART enrollment (declined by 111 people/week relative to baseline). Mean weekly levels of ART enrollment did not decline significantly from the COVID-19 emergency to the start of the hospital strikes. Visually, this null effect appears to be driven by offsetting increases in the latter part of 2020. In contrast, mean levels of enrollment in all other periods were significantly lower relative to baseline. For example, the period following the fuel protests saw a decline of 159 people/week from baseline. Even so, the visual trend lines in each segment following a major disruption was characterized by a pattern of “rebounding” enrollment. Results within the PAP area were similar, with the exception of the period between the fuel protests and intensifying gang violence, where ART enrollment appeared to surge and subsequently decline. However, this decline may have stabilized in the latter part of 2024. Figures for PAP, outside of PAP, and each clinical EMR (along with tables with regression estimates for each EMR) operating in Haiti are provided in the Supplemental Appendix files.

**Table 2:** Estimates of immediate, level, and trend effects from autoregressive integrated moving average models for weekly counts of antiretroviral treatment enrollment in Haiti, PAP, and outside of PAP.

Statistical Effect Type	Effect Description	Haiti		PAP		Outside of PAP	
		Estimate	Sig.	Estimate	Sig.	Estimate	Sig.
	Intercept	**244.61**	***	**43.32**	***	**199.08**	***
Immediate	COVID-19 (week 11, 2020)	−7.28	–	−1.04	–	−26.51	–
	Hospital strikes (week 47–48, 2020)	15.70	–	−11.42	–	17.07	–
	Assassination (week 27–28, 2021)	−14.60	–	−10.15	–	1.80	–
	Fuel protests (week 37–44, 2020)	**−111.10**	**	**−20.81**	*	**−90.53**	**
	Gang violence (week 9–10, 2024)	−50.07		−13.03	–	−32.90	–
Level	COVID-19 to hospital strikes	−46.75		−9.13	–	−40.02	–
	Hospital strikes to assassination	**−98.33**	**	**−27.19**	**	**−77.25**	**
	Assassination to fuel protests	**−110.88**	**	**−33.55**	**	**−83.11**	*
	Fuel protests to gang violence	**−159.11**	**	−26.45	–	**−128.92**	**
	Gang violence to end of study period	**−149.29**	*	**−60.12**	**	−108.31	–
Trend	Overall trend	**0.55**	*	0.11	–	**0.48**	*
	Fuel-to-gang period	**−3.07**	*	**−0.38**	*	**−2.74**	**

ARIMA = autoregressive integrated moving average models; PAP = Port-au-Prince; Sig. = Significance. ARIMA specifications: Haiti = ARIMA (1,0,0), PAP = ARIMA (2,0,1), Outside of PAP = ARIMA (1,0,2). *P <0.05, **P <0.01, ***P <0.0001. Bolding refers to any significance level.

**Figure 2.**
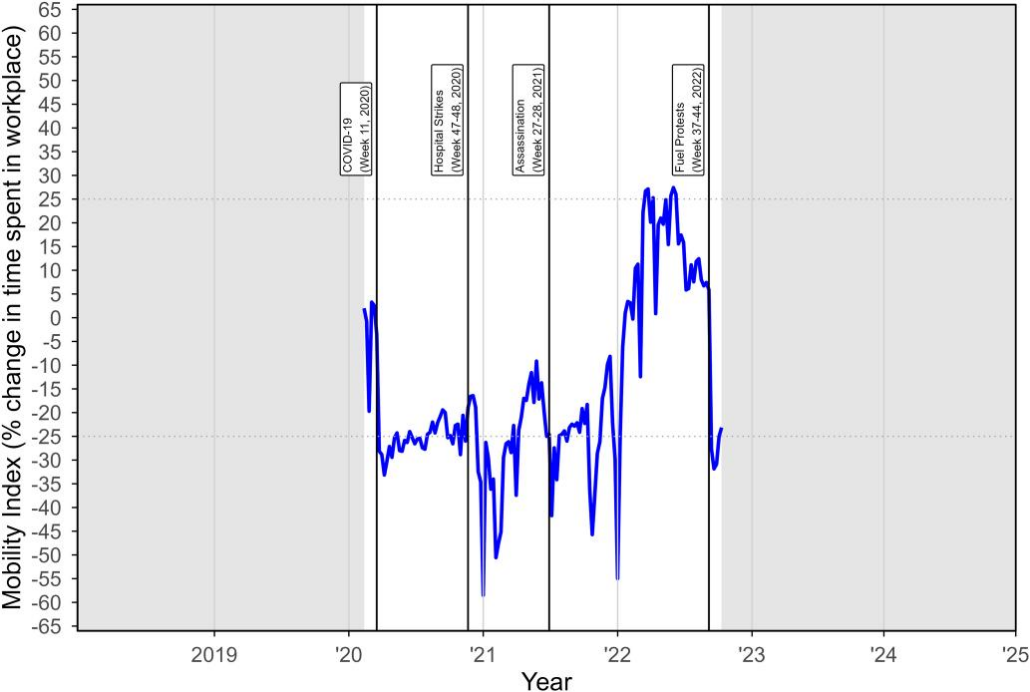
Weekly cell phone mobility index for Haiti, March 2020 to October 2022 (last month available from Google). The trend line represents the percentage change in time spent in the workplace relative to a baseline of zero at the start of the COVID-19 emergency period.

At the start of the pandemic, there was an immediate ∼25% decrease in time spent in the workplace in Haiti, followed by sharp declines after the hospital strikes (see Figure 2). Periods of variability in returning to the workplace are apparent leading up the presidential assassination, but the trend was generally increasing. Another sharp decline was noted after the presidential assassination, with distinct end-of-the-year declines. Just prior to the fuel protests, the population returned to spending approximately 20% more time in the workplace but dropped back to COVID-19 emergency period levels.

## DISCUSSION

As of the end of 2024, that national HIV surveillance system indicates that weekly enrollment in ART in Haiti had declined by 11.5% since 2018. Given the sociopolitical upheaval occurring since the COVID-19 emergency period and an estimated 80% of hospitals and clinics closed in the aftermath of the presidential assassination,^33^ it is remarkable that this decline was not more severe.^5^^,^^20^^,^^33^ Likewise, it is arguably a public health success that the SALVH surveillance system itself has remained resilient enough to confidently examine these trends. There is anecdotal evidence that SALVH even played a role in finding alternative HIV clinics for patients away from affected clinics.^7^^,^^34^ Even after one of the most impactful shocks to the system—the fuel protests in 2022—ART enrollment counts continued a historical pattern of “recovery.” It is not clear whether the increased gang violence in early 2024 will be the mark of declining ART enrollment as indicated by our visual findings around that time. Therefore, we caution against overinterpretation until the 2024/2025 data can be finalized and confirmed.

GHESKIO operates two clinics, located primarily in the capital, that appeared to weather the disruptive events that we modeled despite persistent blockades by gangs and losing more than 50% of its staff.^30^ GHESKIO shifted to a community-based HIV services model during this period, but facility visits still represent the majority of all visits, especially among patients newly initiated on ART.^30^ Still, it is concerning that these facilities have reached historically low levels of newly initiated ART enrollment in recent years. GHESKIO’s substantial decrease in ART enrollment recently, including the sharpest proportional decline among the EMR systems studied, highlights significant ongoing vulnerabilities in maintaining consistent HIV care delivery within highly urbanized and politically unstable contexts.

PIH clinics had ART enrollment rates that increased throughout the study period, eventually surpassing GHESKIO in weekly ART initiations, for the first time, by 2024. PIH primarily operates clinics in the Central Plateau, which, because of their rural location, may have contributed to relative stability and possibly absorbed patients who fled the urban turmoil in PAP. The stable performance of PIH clinics underscores the potential effectiveness of decentralized and rural-focused health care models in maintaining ART services amidst national disruptions occurring in urban areas.

Examining Google’s cell phone mobility data offers additional insight that the events we selected as likely to affect HIV services were truly impactful. Our event selection was highly coincident with very large drops in workplace attendance, which indicates that people stayed home to avoid personal risks. Still, there were other conspicuous declines in weekly workplace mobility around the end of the CY2021 and CY2022, which suggests cyclical effects like holidays and vacations. We could not account for all variability in our analysis, but this type of mobility data is valuable and offers hypothesis-generating potential that could be followed by rigorous testing using public health data.

Overall, our analysis reveals both concerning trends and areas of resilience within Haiti’s HIV service provision landscape. One of the critical questions left unanswered by our analysis is whether declines in ART enrollment are more reflective of the cumulative damage to the capacity of the health care system from these shocks or whether the number of new HIV cases are truly declining (which would be a public health success). One of the strengths of our study is in characterizing SALVH trends over a long period of time in Haiti to help clarify this question and avoid confounding health systems effects with epidemiological realities. For example, in March 2025, UNAIDS warned of a potential increase of 30–50% in new HIV infections if preexposure prophylaxis services were halted as a result of the US President’s Emergency Plan for AIDS Relief (PEPFAR) funding cuts.^35^ Understanding the temporal variability in ART enrollments using this study’s findings as context will help us evaluate whether that prediction is accurate once the 2025 data have stabilized. The insights gained from this study emphasize the importance of adaptive health care delivery models, robust surveillance systems, and the use of novel data sources like mobility trends to inform responsive public health strategies during periods of health and sociopolitical challenges.

### Limitations.

Our study has several limitations. We did not have a standardized approach to selecting disruptive events, and the sheer scale of upheaval means that others may likely be unaccounted for. Furthermore, we did not attempt to explain every change point in our series, some of which could have important public health implications. For example, there appears to be a large increase in ART enrollment from January 1, 2020, up to the beginning of the COVID emergency period for Haiti. The ART enrollment data covering quarterly counts up to Q1 2020 from PEPFAR reporting show an anomalous decrease in Q1 2019, with a rebound by the end of the year.^36^ Our ability to verify counts and clinic closures directly with the EMRs was limited. We were able to compare our counts from SALVH and publicly available reports from Haiti’s National HIV program dashboard at https://salvh.mesi.ht/radar/index.html. For our study period, the RADAR dashboard reported 104,050 newly enrolled ART patients in comparison with our assessment of 94,112 (see Table 1). These differences could result from delays or inconsistencies in facility-level reporting, potential errors in weekly or annual aggregation processes, and/or differences in deduplication methods between the RADAR and SALVH systems. As another cross-check, we examined the new ART initiation counts from SALVH for GHESKIO for time frames reported by Liautaud et al.,^37^ which were very similar (2,217 versus 2,158, respectively). We do not believe these absolute differences materially affect our results and show that SALVH is aligned as expected. Finally, our analysis only considers ART enrollment (i.e., an HIV diagnosis) but not whether patients were retained in care.

## CONCLUSION

Our findings clearly indicate that new ART enrollments significantly declined in Haiti from 2018 to 2024 through pandemic-related and sociopolitical disruptions. Despite the resilience observed in certain clinical systems, the sustained decrease in enrollment, particularly at urban-based facilities, highlights potential ongoing vulnerabilities in HIV service provision. Given these challenges, continued investment from international funding sources, such as PEPFAR, remains critical. Recent funding cuts implemented through the United States Agency for International Development, one of the primary agencies responsible for executing PEPFAR’s programs in Haiti alongside the US Centers for Disease Control and Prevention and the US Department of Defense, further underscore the urgent need for strategic planning, diversified health care delivery models, and robust monitoring systems to mitigate future disruptions and sustain ART services in Haiti. Without a proactive, coordinated response, progress toward HIV epidemic control in Haiti risks significant setbacks.

## Supplemental Materials

10.4269/ajtmh.25-0491 Supplemental Materials
