# Supplementary material for: Trends and Disruptions in Antiretroviral Treatment Enrollment in Haiti, 2018–2024
Source: Am J Trop Med Hyg. 2026 Mar 31;114(5):966–72. doi: 10.4269/ajtmh.25-0491 (PMC13153583; doi:10.4269/ajtmh.25-0491)
Supplement: Supplemental Materials [file tpmd250491.SD1.pdf]

		GHESKIO		iSanté		PIH	
		Estimate	Sig.	Estimate	Sig.	Estimate	Sig.
Effect Type	Intercept	**22.68**	***	**182.59**	***	**30.72**	***
Immediate	COVID-19 (Week 11, 2020)	6.79		-21.29		-2.76	
	Hosp. Strikes (Week 47-48, 2020)	-2.19		9.35		3.54	
	Assassination (Week 27-28, 2021)	-1.02		-20.12		0.89	
	Fuel Protests (Week 37-44, 2020)	-6.22		**-101.09**	**	-5.23	
	Gang Violence (Week 9-10, 2024)	-4.49		-14.11		-7.62	
Mean	COVID-19 to Hosp. Strikes	-3.90		-40.11		-13.81	**
	Hosp. Strikes to Assassination	-4.96		**-88.86**	**	**-22.93**	***
	Assassination to Fuel Protests	-1.31		**-106.01**	**	**-27.32**	***
	Fuel Protests to Gang Violence	-0.22		**-120.81**	**	**-33.03**	***
	Gang Violence to end of study period	-3.80		**-162.73**	**	**-34.75**	**
Trend	Overall trend	-0.03		**0.63**	**	**0.10**	**
	Fuel-to-gang period	n/a		**-1.16**	*		
	Gang-to-end of study period			**-2.68**	**		

Table S1. Estimates of immediate, level, and trend effects from autoregressive integrated moving average (ARIMA) models for weekly counts of antiretroviral treatment enrollment for iSanté; the Haitian Group for the Study of Kaposi's Sarcoma and Opportunistic Infections (GHESKIO); and Partners in Health (PIH) - Zanmi Lasante. p< 0.0001 ***, p< 0.01 **, p < 0.05 *

GHESKIO=ARIMA(2,0,1), iSanté=ARIMA(1,0,0), PIH=ARIMA(2,0,1)

Alt Text: A table showing estimates from a time series model of the immediate and trend effects of socially disruptive events in Haiti by clinical electronic medical record (EMR).

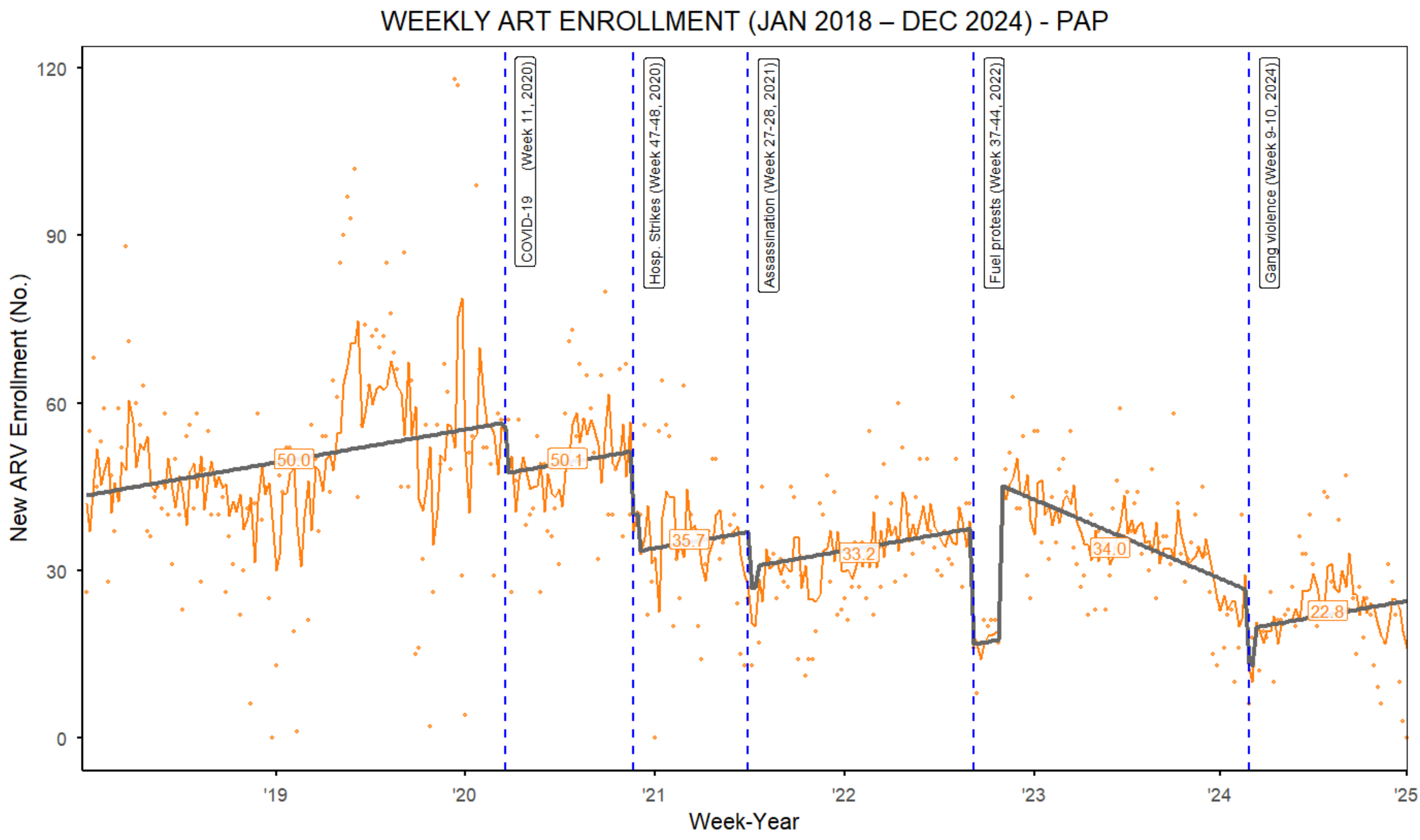

WEEKLY ART ENROLLMENT (JAN 2018 – DEC 2024) - PAP
New ARV Enrollment (No.)
Week-Year
120
90
60
30
0
'19
'20
'21
'22
'23
'24
'25
COVID-19 (Week 11, 2020)
Hosp. Strikes (Week 47-48, 2020)
Assassination (Week 27-28, 2021)
Fuel protests (Week 37-44, 2022)
Gang violence (Week 9-10, 2024)
50.0
50.1
35.7
33.2
34.0
22.8

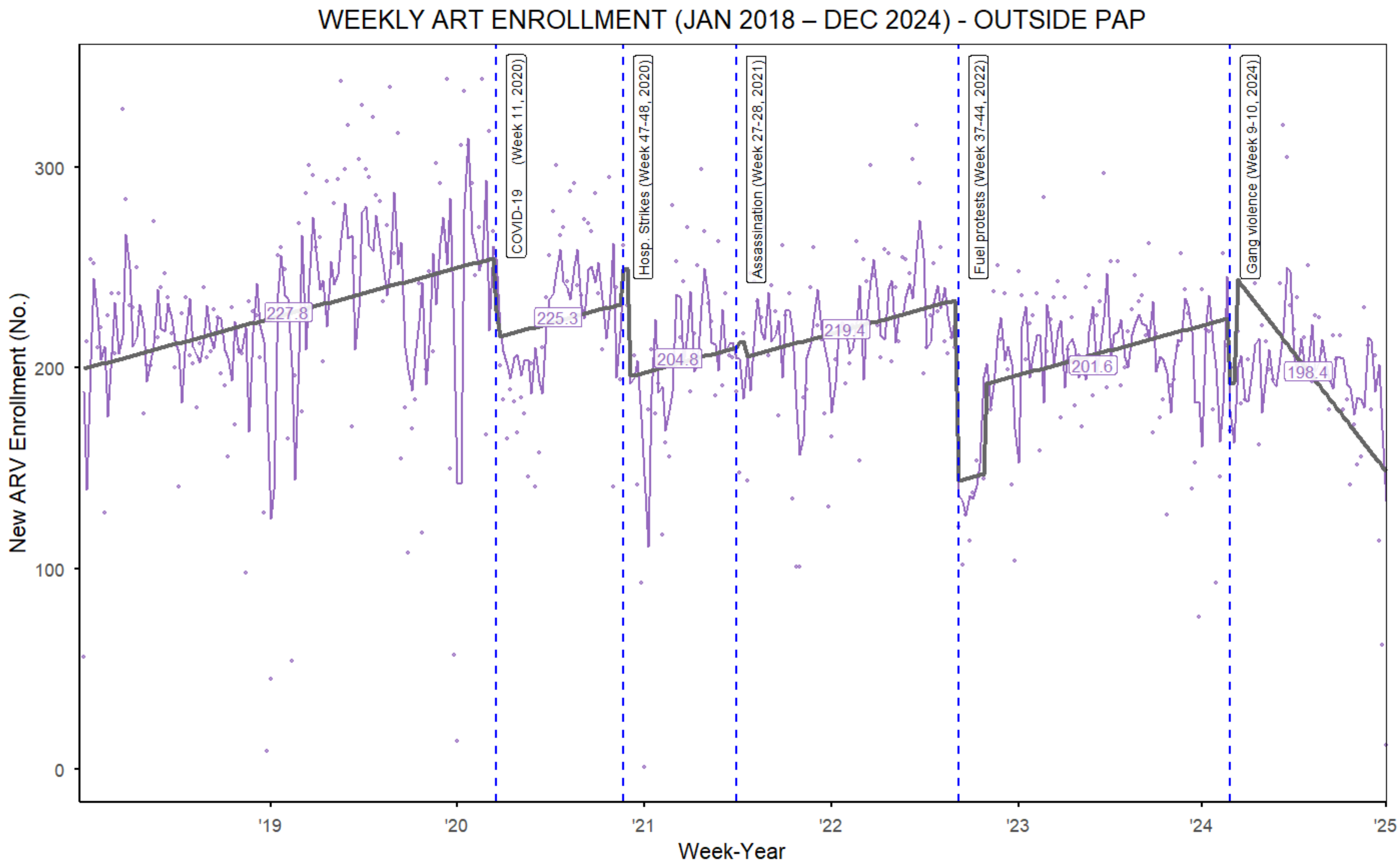

WEEKLY ART ENROLLMENT (JAN 2018 – DEC 2024) - OUTSIDE PAP
New ARV Enrollment (No.)
Week-Year
0
100
200
300
'19
'20
'21
'22
'23
'24
'25
COVID-19 (Week 11, 2020)
Hosp. Strikes (Week 47-48, 2020)
Assassination (Week 27-28, 2021)
Fuel protests (Week 37-44, 2022)
Gang violence (Week 9-10, 2024)
227.8
225.3
204.8
219.4
201.6
198.4

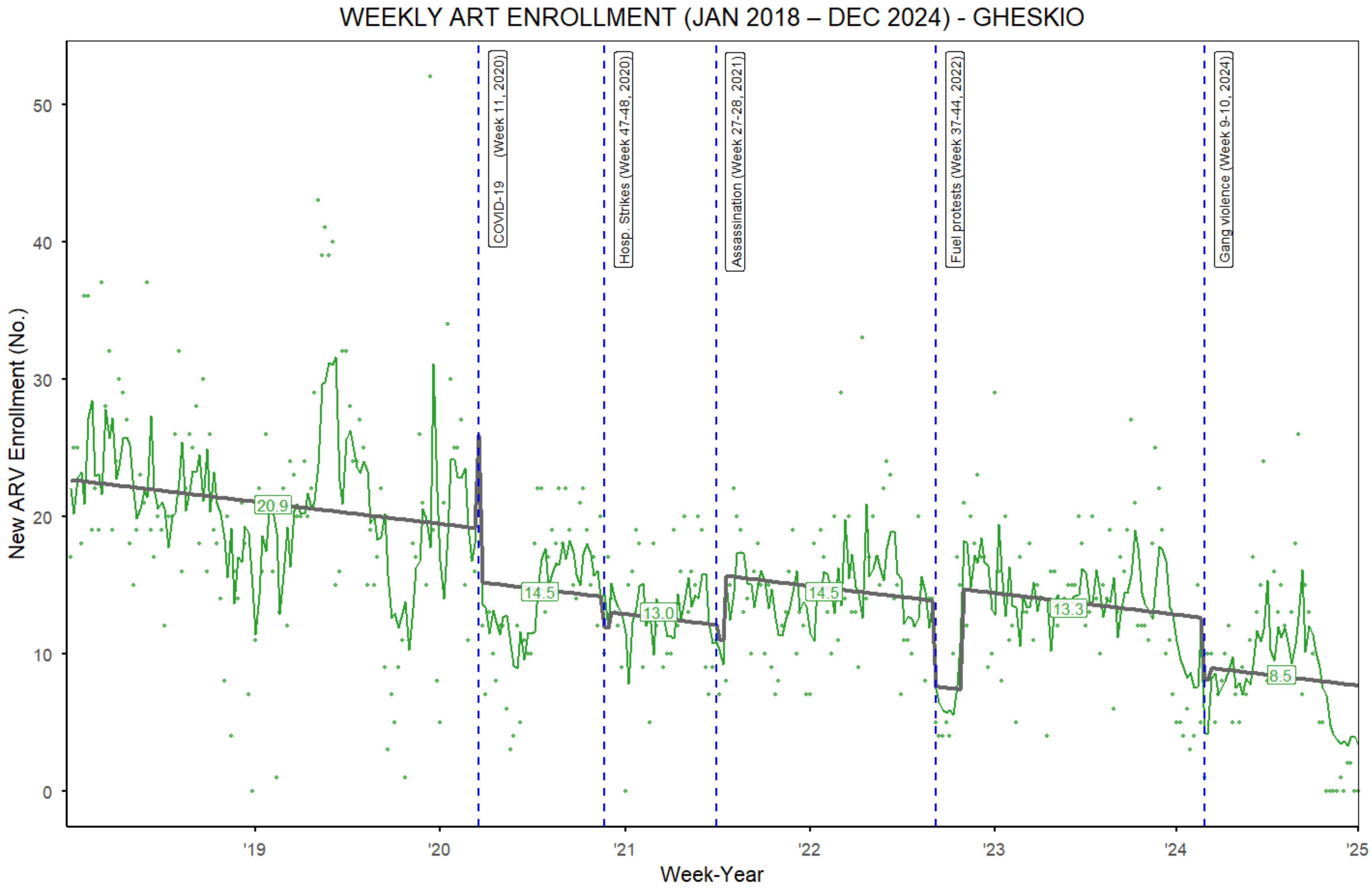

WEEKLY ART ENROLLMENT (JAN 2018 – DEC 2024) - GHESKIO
New ARV Enrollment (No.)
Week-Year
0
10
20
30
40
50
'19
'20
'21
'22
'23
'24
'25
COVID-19 (Week 11, 2020)
Hosp. Strikes (Week 47-48, 2020)
Assassination (Week 27-28, 2021)
Fuel protests (Week 37-44, 2022)
Gang violence (Week 9-10, 2024)
20.9
14.5
13.0
14.5
13.3
8.5

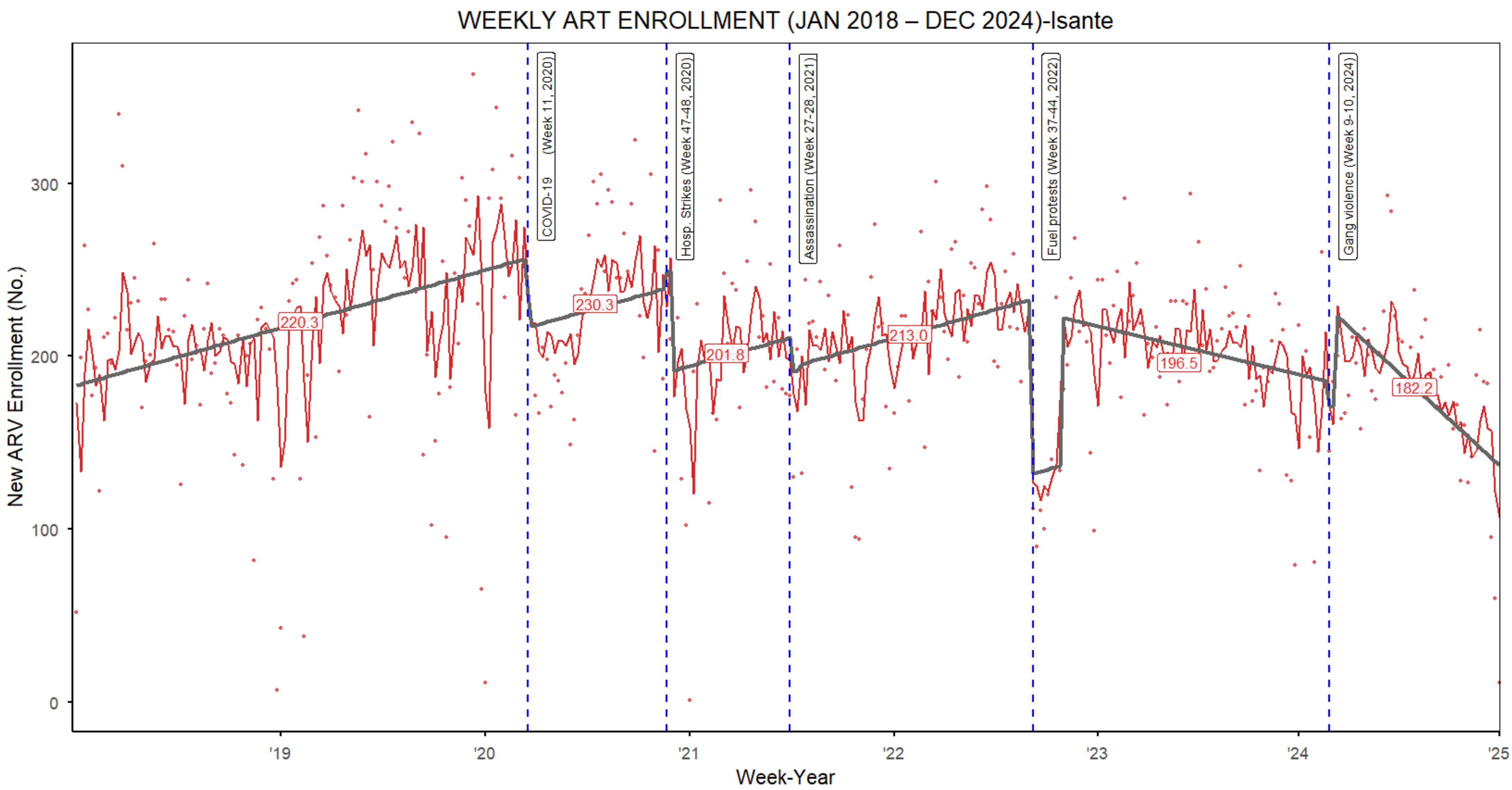

WEEKLY ART ENROLLMENT (JAN 2018 – DEC 2024)-Isante
New ARV Enrollment (No.)
Week-Year
COVID-19 (Week 11, 2020)
Hosp. Strikes (Week 47-48, 2020)
Assassination (Week 27-28, 2021)
Fuel protests (Week 37-44, 2022)
Gang violence (Week 9-10, 2024)
220.3
230.3
201.8
213.0
196.5
182.2
0
100
200
300
'19
'20
'21
'22
'23
'24
'25

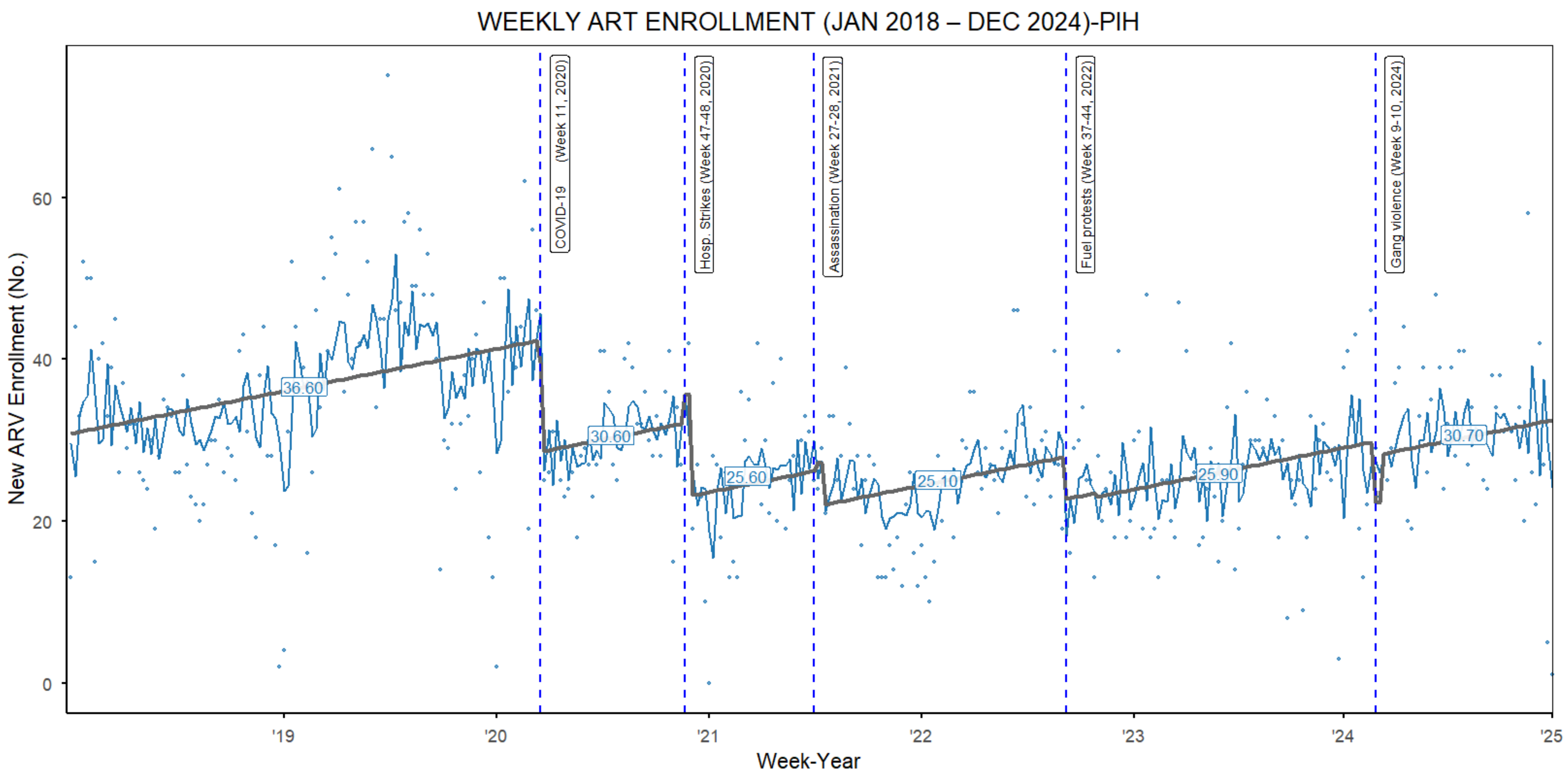

WEEKLY ART ENROLLMENT (JAN 2018 – DEC 2024)-PIH
New ARV Enrollment (No.)
Week-Year
COVID-19 (Week 11, 2020)
Hosp. Strikes (Week 47-48, 2020)
Assassination (Week 27-28, 2021)
Fuel protests (Week 37-44, 2022)
Gang violence (Week 9-10, 2024)
36.60
30.60
25.60
25.10
25.90
30.70
0
20
40
60
'19
'20
'21
'22
'23
'24
'25
